# Temporomandibular arthropathies: A retrospective study with histopathological characteristics

**DOI:** 10.4317/medoral.22739

**Published:** 2019-08-18

**Authors:** Tamires-Aparecida S. Rennó, Amy-Chwen-Jing Chung, Hans-Albrecht Gitt, Luciana Corrêa, João-Gualberto C. Luz

**Affiliations:** 1 Department of Oral and Maxillofacial Surgery, School of Dentistry, University of São Paulo, Brazil; 2 Department of General Pathology, School of Dentistry, University of São Paulo, Brazil; 3 International College for Maxillo-Facial Surgery, Leipzig, Germany

## Abstract

**Background:**

To investigate the incidence of temporomandibular arthropathies diagnosed in a university center and to describe their histopathological characteristics.

**Material and Methods:**

Temporomandibular arthropathy cases with corresponding slides were selected from an oral and maxillofacial surgical pathology service. Cases of exclusively articular disc disease were not included.

**Results:**

The mean age was 31.3 years with a predominance of females (69.7%). Of these diagnoses, 53.6% were unilateral condylar hyperplasia, 17.8% were bony ankylosis, 14.3% were degenerative joint disease, 10.7% were osteochondroma, and 3.6% were synovial chondromatosis. Condylar hyperplasia presented as thick fibrocartilage and cartilage nests in the cancellous bone. Bony ankylosis exhibited lamellar bone and nests of chondrocytes. Degenerative joint disease presented as an irregular layer of fibrocartilage with areas of clustered chondrocytes and calcified cartilage. Osteochondroma of the condyle exhibited hyaline cartilage and areas of new bone formation. Synovial chondromatosis presented as immature cartilaginous tissue and randomly arranged chondrocytes.

**Conclusions:**

The pathological alterations verified in these arthropathies involved diseases that were predominantly proliferative, i.e., unilateral condylar hyperplasia, osteochondroma and synovial chondromatosis of the tumor or pseudotumor type and bony ankylosis associated with callus formation of the reparative type, and less frequent degenerative changes for which the disease is so named.

** Key words:**Temporomandibular joint, pathology, ankylosis, pathology, arthritis, degenerative, osteochondroma, chondromatosis, synovial.

## Introduction

Temporomandibular joint (TMJ) arthropathies occur in the population but are not frequent pathological changes. Arthropathy is a collective term for any disease of the joints. Case series studies of specific arthritis of the TMJ are found in the literature, but there are no reports of the incidence of TMJ arthropathies combined with histopathological characteristics of various arthropathies.

Unilateral condylar hyperplasia is a relatively rare condition that is characterized by a slow-developing, progressive enlargement of the condyle that affects facial symmetry and occlusion (1,2). This disorder is self-limiting. However, while the condition remains active, the asymmetry progresses together with the associated occlusal disturbances (3).

TMJ bony ankylosis is a severe disease that destroys the joint itself, causes hypomobility of the jaw, and affects the functions of speech, mastication and oral hygiene (4). The primary cause of TMJ bony ankylosis is trauma, but this condition can also result from infection (5,6). While there are many publications regarding the treatment of TMJ bony ankylosis, its pathogenesis remains poorly described (7), and protein-energy malnutrition should be considered as a predisposing cause of this entity (8). TMJ bony ankylosis frequently begins during the active growth stage of childhood and results in facial deformity, malocclusion and sleep apnea syndrome (4,7).

A study of the distribution of diagnoses in a population of patients with temporomandibular disorders revealed that 22.3% of cases involved osteoarthritis and osteoarthrosis, which are degenerative joint diseases (9). According to the Research Diagnostic Criteria for Temporomandibular Disorders (RDC/TMD), muscle disorders (Group I) are more frequent, whereas disc displacements (Group II) and arthralgia, arthritis and arthrosis (Group III) are less prevalent. These findings may explain the relative scarcity of studies involving these latter disorders (10). The absolute indication for surgical treatment of osteoarthritis of the TMJ is the presence of focal joint pain that affects the quality of life and a definite pathologic lesion on imaging (11).

Tumors of the temporomandibular joint are infrequent but occur in young adults. Given the limited specificity of the symptoms, i.e., pain, swelling and limited joint movements, many tumors are initially diagnosed as temporomandibular disorders (12). When a patient presents with progressive facial asymmetry, a clinical evaluation and imaging techniques are indicated, and the clinical hypothesis regarding the diagnosis includes condylar hyperplasia, osteochondroma, chondroblastoma, osteoma, osteoblastoma and chondrosarcoma (13). Synovial chondromatosis is classified as a tumorous lesion of the tendofascial tissues that belongs to the group of extraskeletal and chondrogenic neoplasms. The terms synovial chondrometaplasia, osteochondromatosis and synovial chondrosis are used synonymously to describe this entity (14).

The purpose of this study was to investigate the incidence of temporomandibular arthropathies diagnosed in a university center and provide descriptions of the associated histopathological characteristics.

## Material and Methods

The cases were selected from histopathological reports from the Oral and Maxillofacial Surgical Pathology Service of the University of São Paulo - USP from 2000 to 2010. Clinical data were collected from stored biopsy reports. This study received approval from the local human research ethics committee (Process CAAE: 01464212.0.0000.0075).

The inclusion criteria included cases, regardless of age, race or gender, who presented with a history of temporomandibular joint surgery due to arthropathies and had corresponding specimens that had been sent for histopathological analysis. Only cases with available hematoxylin and eosin (H & E) - stained slides that were well preserved were accepted. The exclusion criteria were cases with incomplete data or histological slides that were inadequate for microscopic evaluation. Cases exclusively involving articular disc displacement or disc tissues were not included. Regarding the Research Diagnostic Criteria for Temporomandibular Disorders (RDC/TMD), only cases that were considered to involve temporomandibular disorders comprising arthralgia, arthritis and arthrosis (Group III) were included (10). The slides were evaluated under light microscopy, and findings regarding the phenomena that occurred within the temporomandibular joint or the condyles were recorded.

Age, gender, ethnic group and unilateral or bilateral occurrence were recorded. Regarding histopathological evaluations of the specimens, the following criteria that have been described in other studies were used according to the type of arthropathy: unilateral condylar hyperplasia (15-17), bony ankyloses (7,18,19), degenerative joint disease (20-23), osteochondroma (13,24), and synovial chondromatosis (25). For cases of condylar hyperplasia, the lesions were classified based on the system of Slootweg and Müller (15). This classification identifies four histologic types, which are described in detail in Villanueva-Alcojol *et al.* (26). Two experienced pathologists and two research fellows conducted the slide review.

The data were collected and entered into a Microsoft Excel spreadsheet (Microsoft Corporation, Redmond, WA, USA). Once the data were tabulated, descriptive statistics were performed.

## Results

There were 53 cases of temporomandibular arthropathies among these files. Twenty-eight cases of temporomandibular arthropathies with the corresponding slides were included in this study according to the aforementioned criteria. After review of the slides, the diagnoses of four cases (cases No. 22, 23, 30 and 31; 12.1%) were changed. Interestingly, all of these cases had clinical diagnoses of bony ankyloses, received histopathological diagnosis of articular tissue fragment (two cases) or condyle without cartilage (two cases) that was partially confirmed. The new diagnoses were bony ankyloses (two cases), unilateral condylar hyperplasia (one case) and degenerative joint disease (one case). 

According to the histopathological diagnoses, unilateral condylar hyperplasia was predominant (n=15; 53.6%) followed by bony ankylosis (n=5; 17.8%), degenerative joint disease (n=4; 14.3%), osteochondroma (n=3; 10.7%) and synovial chondromatosis (n=1; 3.6%). The unilateral condylar hyperplasia cases involved eight juvenile cases and seven adult cases. Of the five cases of bony ankylosis, four were unilateral, and one was bilateral.

The mean age was 31.3 years. Among the numerous patients with arthropathies, the interval of 11-20 years of age was predominant followed by the intervals of 21-30 years and 31-40 years. Female patients were more frequent (69.7%) among the patients with all arthropathies. Regarding ethnicity, 26 (78.8%) of the patients were Caucasian, and seven (21.2%) were Afro-American. Among the patients with condylar hyperplasia, 11 (73.3%) were Caucasian, and four (26.7%) were Afro-American. Among the patients with other arthropathies of the TMJ, Caucasians predominated.

In the review of the microscope slides, the following specific histopathologic findings that are characteristic of the main arthropathy were identified. 

**Figure 1:**
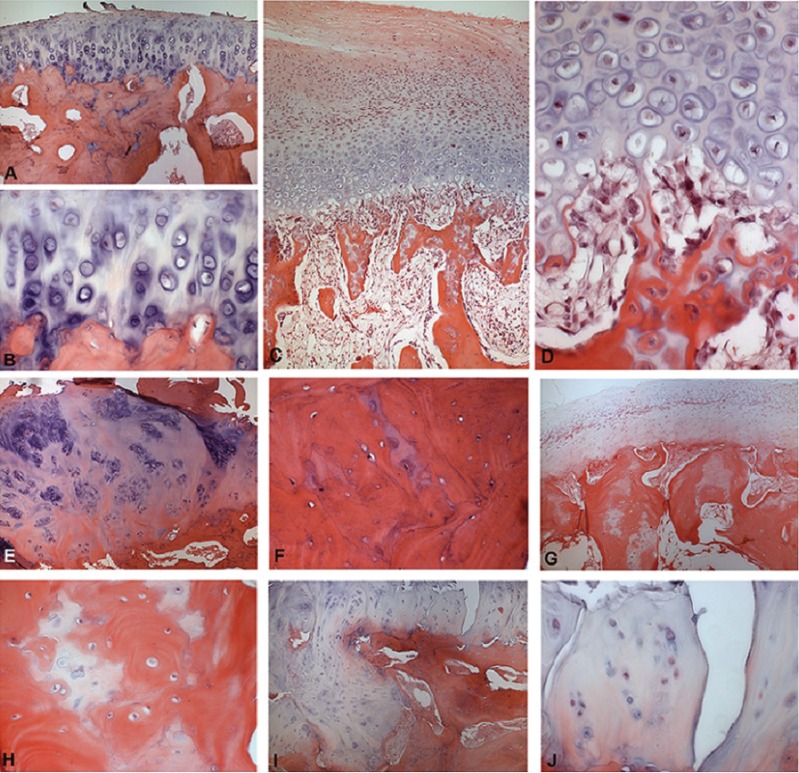
Representative histological sections for juvenile (A–D) and adult (E–J) condylar hyperplasia (hematoxylin and eosin, 100X and 400X original magnification). Juvenile - A: Hyaline cartilage exhibiting a predominant pattern of hypertrophic zone. B: Detail of previous case showing large chondrocytes surrounded by basophilic matrix adjacent to the bone. C: Hyperplasia of growth cartilage with thick and irregular proliferative and resting zones. D: Detail of trabecular zone of the previous case showing high frequency of osteoclasts. Adult - E: Hyperplasia of hyaline cartilage tissue on condylar surface showing a great amount of intercellular matrix and isogenous group of chondrocytes with an irregular pattern scattered in the tissue. F: Detail of the previous case exhibiting osteoid foci in the trabecular bone. G: Thick and hypercellularized fibrocartilage with eosinophilic matrix covering the entire condylar surface. H: Detail of the previous case showing foci of cartilage in the trabecular bone. I: Irregular condylar surface covered by a hyperplastic hyaline cartilage. J: Detail of the previous case focusing an area of few chondrocytes and great amount of intercellular matrix originating a discontinuous layer.

Among the cases of condylar hyperplasia, characteristics of the juvenile and adult forms were found. Among the juvenile form cases, the following histopathologic features predominated: a hyaline cartilage layer with hypertrophic chondrocytes, a well cellularized proliferative zone, a continuity of the cartilage with the interior of the trabecular bone, and a thick layer of fibrocartilage (Fig. 1 A-D). In the adult form, the following characteristics were observed: clustering of the chondrocytes, cartilage foci within the trabecular bone, a thick layer of fibrocartilage or a layer of varying thickness, and the presence of clefts in the fibrocartilage ( Table 1) (Fig. 1 E-J). The condylar hyperplasia cases were classified based on the histologic types established by Slootweg and Müller (15), and the most predominant type was type I.

**Table 1:**
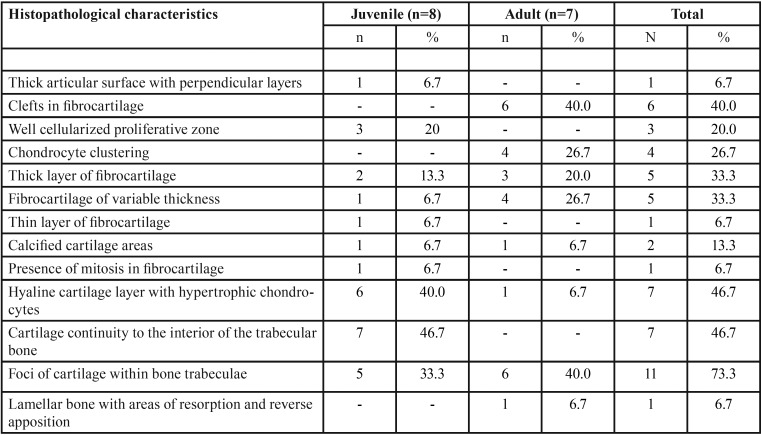
Frequency of the main histopathological findings observed in juvenile (n = 8) and adult (n = 7) condylar hyperplasia cases (n=15).

**Figure 2:**
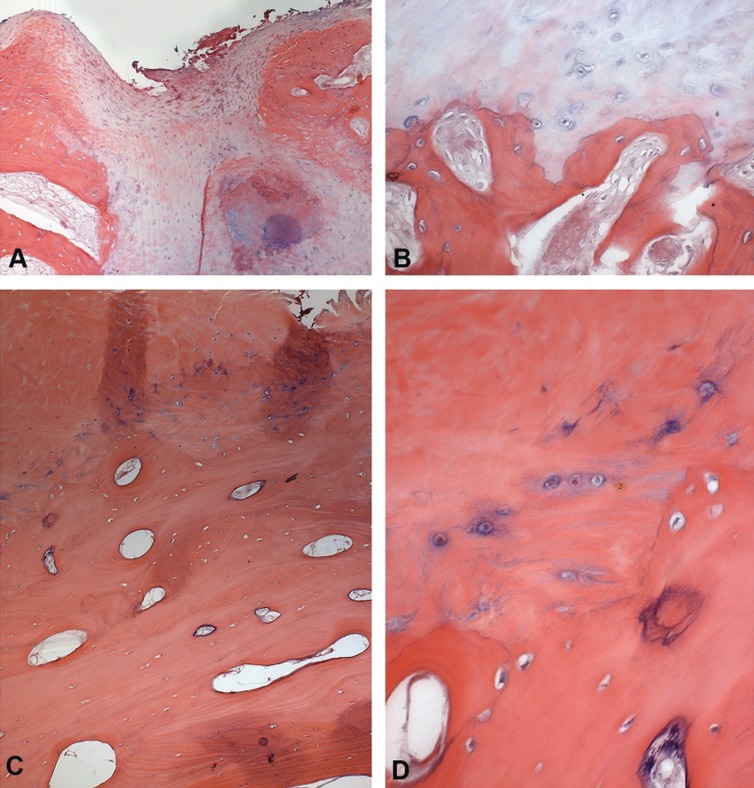
Representative histological sections for bony ankylosis (hematoxylin and eosin, 100X and 400X original magnification). A: Presence of cartilage in the middle of the trabecular bone, which is covered by thick and cellularized connective tissue. B: Detail of the cartilage described in the previous case, which is cell poor and formed by a pale eosinophilic matrix without typical characteristics of cartilaginous matrix. C: Trabecular bone covered by a large layer of hyaline connective tissue. D: Detail of the connective tissue in which some chondrocyte-like cells are seen scattered on the hyaline matrix.

All cases of TMJ ankylosis involved bony ankylosis, and the following characteristics were observed: bone remodeling areas, mature lamellar and cellularized bone tissue, cartilaginous tissue formed by chondrocytes with proliferative activity with presence of nests of chondrocytes, quite compact bone tissue with minimal bone marrow, and thick layers of fibrous connective tissue ( Table 2) (Fig. 2).

**Table 2:**
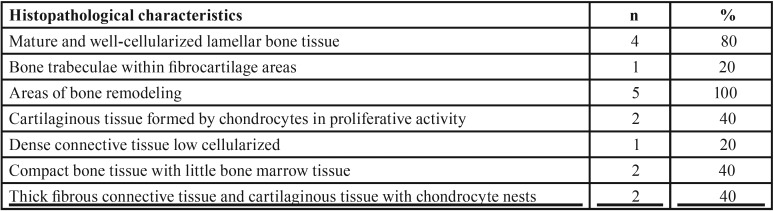
Frequency of the main histopathological findings observed in cases of bony ankylosis.

**Figure 3:**
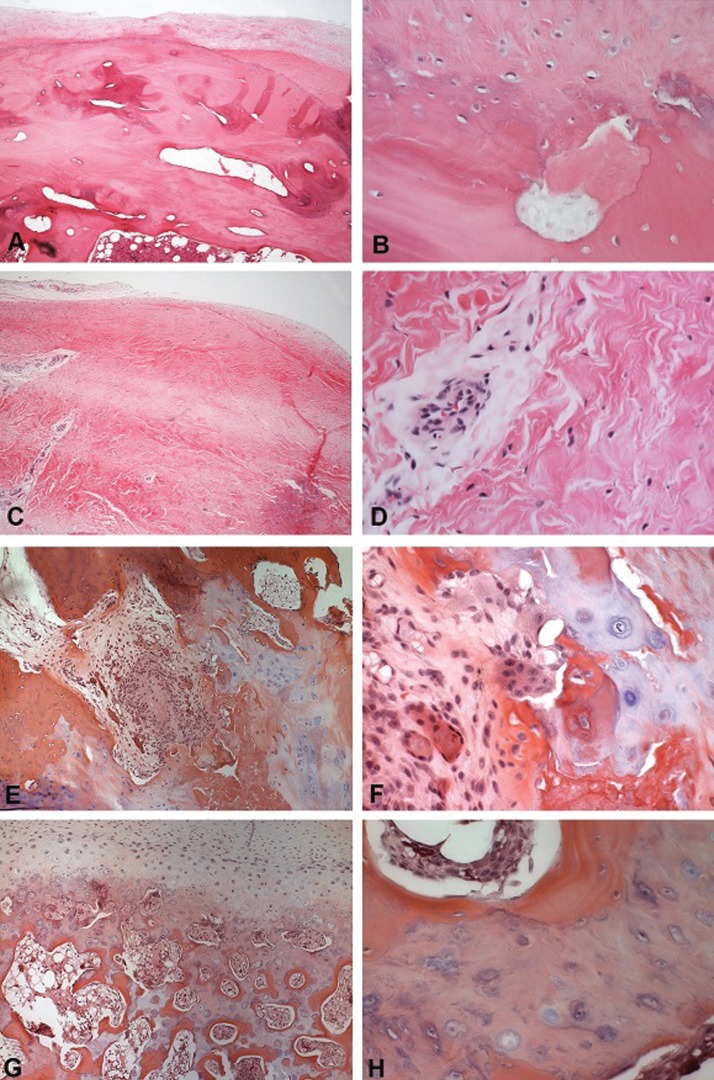
Representative histological sections for degenerative joint disease (A–D) and osteochondroma (E–H) (Hematoxylin and eosin, 50X and 400X original magnification). Degenerative joint disease - A: Presence of irregular layer of cartilage on the articular surface of condyle. B: Detail of the cartilage, showing nonhomogeneous distribution of chondrocytes and islands of cartilage-like tissue in the bone. C: Fibrous connective tissue in the region of articular capsule, exhibiting dense collagen matrix and large blood vessels. D: Few cells were seen in the connective tissue, which showed a hyaline pattern. Osteochondroma - E: Condylar surface showing a mixture of cartilage, bone, connective and necrotic tissue, rich of cells with an irregular distribution. F: Detail of previous case showing cells with a varied phenotype, resembling or chondrocytes or osteoblasts, with hyperchromatic nuclei and evident nucleoli, scattered in fibrous, bone and cartilage-like matrices. G: Condylar surface covered by a thick cartilage rich of large chondrocytes, invading the trabecular bone. H: Detail of the previous case showing cartilage tissue composed of hypertrophic chondrocytes localized in the middle of bone matrix.

The following characteristics were found among the cases of degenerative joint disease: an articular surface with fibrous connective tissue of variable thickness, intense osteoclastic activity with areas of bone neoformation and osteoid deposition, dense connective tissue that was richly hyaline and slightly vascularized, an irregular layer of fibrocartilage with chondrocyte clustering areas, and cartilage that was separated from the subchondral bone by a calcified layer ( Table 3) (Fig. 3 A-D).

**Table 3:**
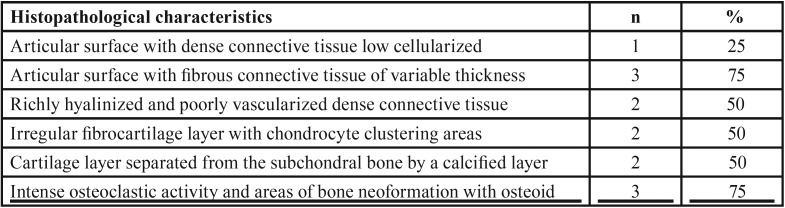
Frequency of the main histopathological findings observed in cases of degenerative joint disease.

The following characteristics were observed among the cases of osteochondroma of the condyle: mononuclear chondrocytes that defined the transition area between the bone tissue and the cartilage, and areas of new bone formation ( Table 4) (Fig. 3 E-H).

**Table 4:**
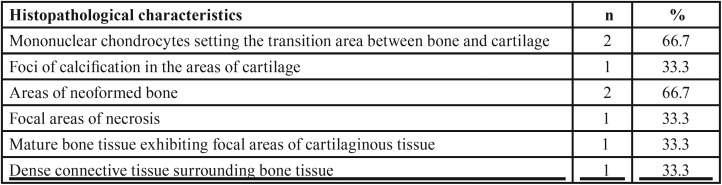
Frequency of the main histopathological findings observed in the cases of osteochondroma of the condyle.

Finally, the following characteristics were observed in the case of synovial chondromatosis: immature cartilaginous tissue that occasionally exhibited a myxoid appearance, disordered cellularity, and randomly arranged chondrocytes.

## Discussion

Temporomandibular joint arthropathies constitute a challenge to oral and maxillofacial surgeons both with regard to diagnosis and surgical treatment. Due to the low frequency of these conditions, population studies are scarce. The present study assessed the incidence of temporomandibular arthropathies that were diagnosed in a university center and provided descriptions of the associated histopathological characteristics to contribute to improved knowledge of these pathological changes, which could subsequently contribute to better treatment being offered to patients with such changes. The study was based on samples from the archives of an oral and maxillofacial surgical pathology service. The possibility that not all of the cases that underwent surgery included surgical specimens that were sent for examination should be considered. However, the incidence of temporomandibular arthropathies was described. 

The demographic features of the study sample included a mean age within the fourth decade of life, a predominance of females and a predominance of Caucasians. The mean age was 31.3 years, and many of the arthropathies were verified to occur predominantly in adolescents and young adults. However, the diagnoses were age dependent, and different frequencies were observed according to the pathological alteration. Unilateral condylar hyperplasia predominated in adolescents and young adults, bony ankyloses predominated in adolescents and adults, and other arthropathies were frequently noted in the adult population. Unilateral condylar hyperplasia is generally observed at a mean age that falls into the third decade of life but the range of ages is wide and includes early adolescents and aged patients (3,26). Bony ankylosis is frequent in children, but young adulthood is the most frequent time of presentation (4,6,7,27). Degenerative joint disease has been reported to be frequent in patients with mean age of 34 years (22), whereas advanced stages of temporomandibular joint degeneration are frequently observed in elderly or aged individuals (9,11,23,28). Osteochondroma has been reported to occur most frequently within the fourth decade of life (12,24). Synovial chondromatosis is most frequent in the fifth decade of life (12,14). 

For most of the arthropathies reported in this study, females predominated. The predominance of females among unilateral condylar hyperplasia cases is known, and females represent approximately 70 - 75% of cases (3,26). It has been suggested that this condition may be a predominantly female condition; however, the possibility that treatment might be more commonly sought by females than males should be considered (1). Moreover, degenerative joint disease is more frequent in females (11,22,28), and hormonal differences could account for this incidence (29). Osteochondroma has been reported to be more frequent in females (12,24). Synovial chondromatosis is also more frequent in females (12,14). However, there is no consensus in the literature concerning bony ankyloses; some studies have reported a male predominance (4,30), and others have observed a female predominance (6). Similar to condylar fractures, the possibility that bony ankylosis is actually more frequent in males should be considered because trauma is the main cause of this condition (30).

The main findings regarding the cases of condylar hyperplasia were the presence of a proliferative zone, hypertrophic chondrocytes, a subchondral bone plate that was not closed, and cartilage rests in the cancellous bone. These observations characterize the activity of the articular cartilage layers and indicate surgical management (1,16,26). A non-uniform picture of the histopathological findings associated with condylar hyperplasia has been described and includes variations in the depth of the cartilage islands and the thickness of the fibrous layer of the articular surface (2). However, the presence of an interrupted layer of undifferentiated mesenchymal cells, a hypertrophic cartilage layer, and cartilage rests in the subchondral spongiosa are characteristic of condylar hyperplasia (26). A comparative study of normal and hyperplastic condyles revealed that the thickness of the hyperplastic cartilage layer is significantly increased in condylar hyperplasia (31). Associations between histologic appearances and joint symptoms have been described, e.g., frequent pain and joint sounds are associated with the presence of a patchy distribution of cartilage layers in which cell-rich areas alternate with nonproliferative cell-poor zones (26). In our study, the cases included both the juvenile and adult forms as initially classified according to the age of onset, and the histopathological findings confirmed this classification (15). In the juvenile form, findings of a hyaline cartilage layer with hypertrophic chondrocytes, a well-cellularized proliferative zone, continuity of the cartilage with the interior of the bone trabeculae, and a thick layer of fibrocartilage, predominated, and these findings are characteristic of this form of condylar hyperplasia (15,17). In the adult form, clustering of the chondrocytes, cartilage foci within the trabecular bone, a thick layer of fibrocartilage or a layer of varying thickness, and the presence of clefts in the fibrocartilage were observed, and these findings are characteristic of this form of condylar hyperplasia (15,17).

The main findings from the ankylosis cases were bone remodeling areas, mature lamellar and cellularized bone tissues, cartilaginous tissue formed by chondrocytes undergoing proliferative activity with the presence of nests of chondrocytes, quite compact bone tissue with reduced amounts of bone marrow, and thick layers of fibrous connective tissue. These findings are characteristic of bony ankyloses, and there were no cases of fibrous ankylosis. Bony ankylosis is the most frequent form of temporomandibular ankyloses (4,6). Many of the cases of bony ankylosis in our series were unilateral, which confirms the reports in the literature (4,6). There are few reports of the histopathological findings associated with temporomandibular ankylosis, and most of these studies were morphological and based on radiological or tomographic descriptions. The concept of an ankylotic mass involves abnormal bone that replaces the articulation and results in a restriction of mandibular movement (18). This bone is capable of continued growth that is associated with a reparative process that is similar to that observed in exuberant calluses that occur in fractures in children (18,32). The presence of bone remodeling areas and mature lamellar and cellularized bone tissue could confirm this hypothesis. Cartilaginous tissue was present in a portion of our cases. Remnants of fibrocartilage, presumably of discal origin, have been reported in many ankylosed temporomandibular joints (27). Thick layers of fibrous connective tissue were verified in a portion of our cases. Previous studies of bony ankylosis have reported that the joint is surrounded by dense fibrous tissue, mainly in the medial aspect (18).

The main findings from the cases of degenerative joint disease were an articular surface with fibrous connective tissue of variable thickness, intense osteoclastic activity and areas of bone neoformation with osteoid deposition, dense connective tissue richly hyaline and slightly vascularized, an irregular layer of fibrocartilage with areas of chondrocytes clustering, and cartilage that is separated from the subchondral bone by a calcified layer. These findings comprise most histological parameters for osteoarthrosis or degenerative joint disease (20-22). A correlation between degenerative changes in the temporomandibular joint and aging has been described and could reflect an accumulation of tissue damage due to a decline in the cellular capacities for adaptation (23,29). 

The main findings related to osteochondroma of the condyle were mononuclear chondrocytes that defined the transition area between bone tissue and cartilage and areas of new bone formation. These findings are characteristic of osteochondroma (13,33). The management of osteochondroma is surgical excision of the tumor, and the diagnosis is based only on clinical and tomographic findings, but the diagnosis has been confirmed by histopathologic examination (12,13).

The main findings regarding synovial chondromatosis were immature cartilaginous tissue with an occasionally myxoid appearance, disordered cellularity, and randomly arranged chondrocytes. These findings are characteristic of synovial chondromatosis (14,25). Synovial chondromatosis has been reported to correspond to the metaplastic transformation of the synovial tissue into chondroid tissue (12).

The main histopathological characteristics of the analyzed arthropathies were confirmed. Moreover, some tendencies regarding the frequencies of temporomandibular joint arthropathies, excluding pathologic alterations of the articular disc, were demonstrated in this study. Thus, in our experience, it can be affirmed that the verified pathological alterations associated with the TMJ arthropathies were predominantly proliferative, i.e., unilateral condylar hyperplasia, osteochondroma and synovial chondromathosis comprised the tumor or pseudotumor type, and bony ankylosis comprised the reparative type, which was associated with callus formation. Degenerative changes were less frequently associated with temporomandibular arthropathies. Therefore, oral and maxillofacial surgeons must consider these findings during the diagnostic evaluation and surgical planning of pathologic temporomandibular joint alterations.
